# Artificial intelligence innovations in neurosurgical oncology: a narrative review

**DOI:** 10.1007/s11060-024-04757-5

**Published:** 2024-07-03

**Authors:** Clayton R. Baker, Matthew Pease, Daniel P. Sexton, Andrew Abumoussa, Lola B. Chambless

**Affiliations:** 1 Vanderbilt University School of Medicine, Nashville, TN USA; 2 Department of Neurosurgery, Indiana University, Indianapolis, IN USA; 3 Department of Neurosurgery, Duke University, Durham, NC USA; 4 Department of Neurosurgery, University of North Carolina at Chapel Hill Hospitals, Chapel Hill, NC USA; 5 Department of Neurosurgery, Vanderbilt University Medical Center, Nashville, TN USA

**Keywords:** Artificial intelligence (AI), Machine learning (ML), Computer vision (CV), Augmented / virtual reality (AR/VR), Neurosurgery, Brain tumor

## Abstract

**Purpose:**

Artificial Intelligence (AI) has become increasingly integrated clinically within neurosurgical oncology. This report reviews the cutting-edge technologies impacting tumor treatment and outcomes.

**Methods:**

A rigorous literature search was performed with the aid of a research librarian to identify key articles referencing AI and related topics (machine learning (ML), computer vision (CV), augmented reality (AR), virtual reality (VR), etc.) for neurosurgical care of brain or spinal tumors.

**Results:**

Treatment of central nervous system (CNS) tumors is being improved through advances across AI—such as AL, CV, and AR/VR. AI aided diagnostic and prognostication tools can influence pre-operative patient experience, while automated tumor segmentation and total resection predictions aid surgical planning. Novel intra-operative tools can rapidly provide histopathologic tumor classification to streamline treatment strategies. Post-operative video analysis, paired with rich surgical simulations, can enhance training feedback and regimens.

**Conclusion:**

While limited generalizability, bias, and patient data security are current concerns, the advent of federated learning, along with growing data consortiums, provides an avenue for increasingly safe, powerful, and effective AI platforms in the future.

## Introduction

**Fig. 1:**
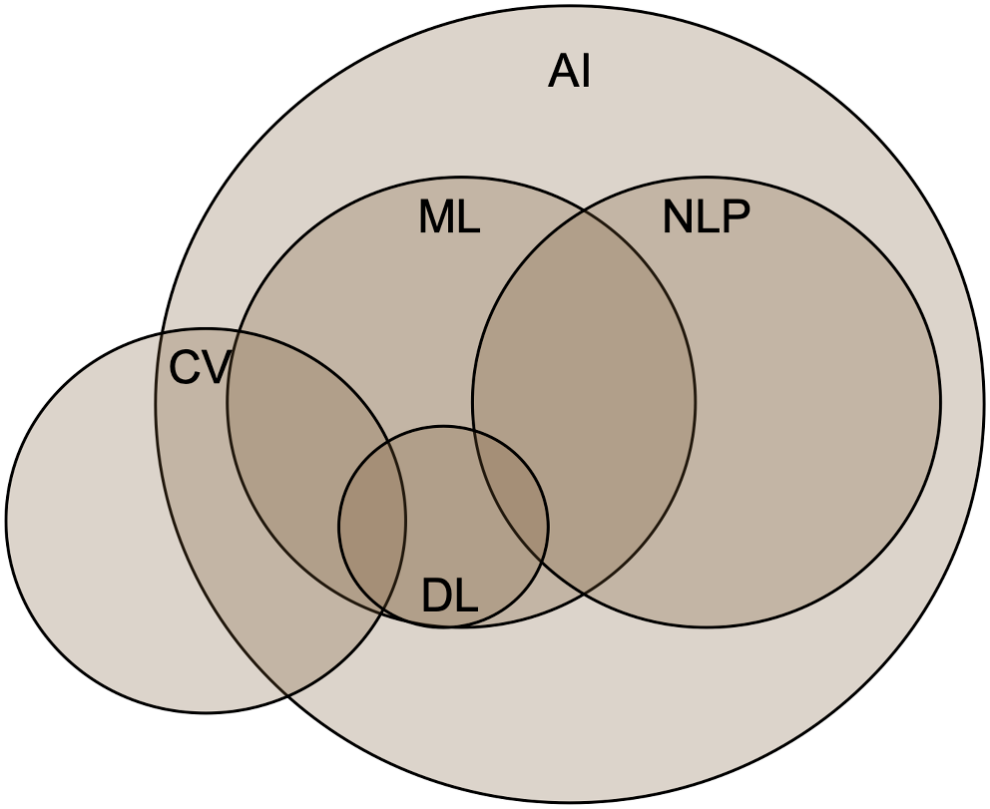
AI is a broad term with many interconnected concepts. While machine learning (ML) and natural language processing (NLP) are separate fields, they both leverage tools from each other, including deep learning (DL). Similarly, computer vision (CV) utilizes ML and DL but also many tools outside of the AI umbrella

Advanced technologies, generally under the umbrella of artificial intelligence (AI), are having a profound impact on the practice of medicine and particularly with neuro-oncological patient care. A brief definition of the different AI components and principals discussed in this review are described below to outline their interconnected relationship (Table 1; Fig. 1). AI is the use of technology to simulate intelligence and human thought [1]. Machine learning (ML) is a subset of AI that uses algorithms to recognize patterns and learn from data [1]. Neural networks, inspired from the brain’s neural structure, are complex interconnected layers of information [1, 2]. Deep learning is a type of ML algorithm that leverages neural networks to iterate its knowledge base and performance [1, 3]. Similar to deep learning, radiomics is another approach that is often used for radiographic analysis. However, it is trained with a pre-defined feature set that generates model variables, and the model can be based in linear regressions or ML algorithms [3]. Computer vision (CV) leverages different imaging hardware (like cameras and microscopes) along with ML and other algorithms to understand and analyze images [1]. Often, CV is used in radiomic feature preparation. Natural language processing (NLP) is a different type of AI that leverages ML (and deep learning) to understand and create text [4]. Augmented reality (AR) superimposes virtual information into the real world, while virtual reality (VR) creates an entirely new world [5]. Importantly, both AR and VR systems utilize AI, including ML and CV, to process and construct these environments [5]. Collectively, these different tools are dramatically improving medicine, especially within neuro-oncology.

**Table 1:** Defining relevant AI concepts

Term	Definition
Artificial Intelligence (AI)	The use of technology to simulate intelligence and human thought [1]
Machine Learning (ML)	A subset of AI that uses algorithms to recognize patterns and learn from data [1]
Deep Learning	A type of ML algorithm based in neural networks to iterate knowledge base and model performance [1, 3]
Neural Networks	Complex, interconnected nodes of information and algorithms inspired by the brain’s neural structure [1, 2]
Computer Vision (CV)	Computerized image analysis that leverages AI, including ML, along with other image capturing hardware (cameras, microscopes, etc.) [1]
Radiomics	An approach to radiographic analysis that harnesses pre-defined feature sets to generate variables, which can power linear regressions or ML models (including deep learning models) [3]
Natural Language Processing (NLP)	A type of AI that utilizing ML, including deep learning, to analyze language and create text [4]
Augmented Reality (AR)	A process that utilizes AI, including ML and CV, to superimpose virtual information into the real world [5]
Virtual Reality (VR)	Like AR, VR also uses ML and CV to create an entirely new, virtual world [5]

**Fig. 2:**
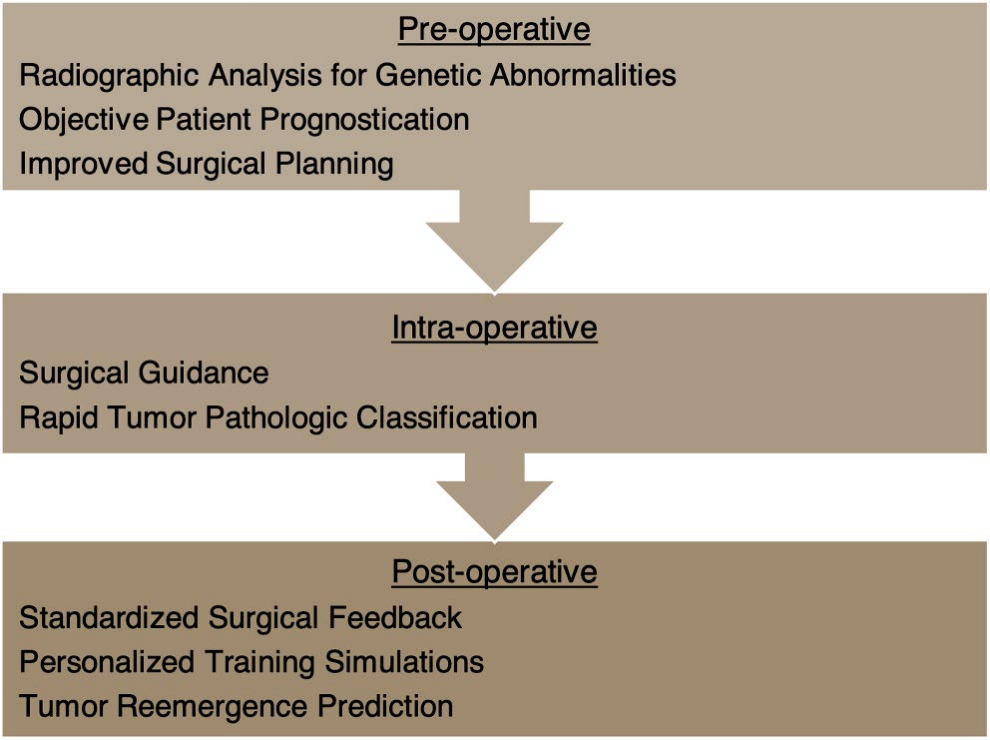
*AI technologies* influence care in a variety of ways throughout a neuro-oncological patient’s experience, from their pre-operative visits to their surgery and beyond

The innovative nature of neurosurgery, along with the recent computing advances discussed above, has led to further clinical integration of impactful AI tools. Brain and spinal tumor treatment, in particular, has benefitted from platforms powered by ML, CV, and AR/VR. From pre-operative prognostication and planning to intra-operative pathologic identification and post-operative surgical feedback, AI is enhancing patient care at every step of neuro-oncologic treatment (Fig. 2). Moreover, AR/VR platforms are creating immersive surgical training supplements, with real-time feedback. In this review, we discuss AI tools currently improving the field of surgical neuro-oncology.

### Preoperative care

A diagnosis of a Central Nervous System (CNS) tumor can be challenging for patients and their families. The initial patient encounter, including obtaining a diagnosis and prognosticating likely outcomes, helps shape the patient’s experience with the healthcare system and can guide their medical decisions. AI is improving this process through harnessing tremendous quantities of data–nearly 1/3 of all data in the world is in the electronic health record (EHR)–and finding associations with pathology and outcomes [6, 7].

Leveraging AI has improved non-invasive, imaging-based diagnosis of CNS tumors, which benefits from rapid, early identification and treatment. Radiomics is a quantitative approach using AI to extract features, or variables, from radiographic images and to use these features to build predictive models [3]. In cranial imaging, multiple studies have demonstrated that radiomic approaches can recognize radiographic abnormalities much more quickly and accurately than radiologists [8–10]. In oncology, these approaches for imaging analysis can identify genetic abnormalities and extract predictive information with a high degree of accuracy [11]. Using this approach, Pease and Gersey et al. employed the Maximum Relevance Minimum Redundancy technique to identify the most relevant glioblastoma features to build a robust predictive pipeline, which estimates MGMT methylation, EGFR amplification, and molecular subgroup [11]. Differentiating tumor pathologies with radiomic models can help predict overall survival and accurately detail tumor genetics [11–13].

While radiomic MRI analysis benefits from expert level feature identification, this process requires a higher time commitment, and deep learning is a more automated approach [11, 14]. Allowing the computer to learn on its own, even identifying features unnoticeable to the human eye, has similarly resulted in powerful pre-operative genetic predictions. Cluceru et al. employed this approach to develop a glioma subtype classifier [15]. This model leveraged traditional T2 images with diffusion weighted images to concurrently evaluate for IDH mutations and 1p19q codeletions [15]. In a similar approach, Shu et al. identified Ki-67 to predict aggressive pituitary adenoma invasiveness [16]. This information can dictate treatment by better outlining available targeted therapies and surgical need. Applying a deep-learning strategy beyond primary tumors, Grossman et al. distinguished between metastasized small cell and non-small cell cancers [17]. Since small cell cancer is usually more aggressive but not typically surgically treated, better differentiating it from non-small cell cancer can vastly alter patient care [17]. Indeed, both radiomics-based and deep learning-based MRI analysis provide a non-invasive, pre-operative characterization of primary and metastasized tumors that leads to better treatment.

Beyond tumor classification, AI tools have led to accurate prognostic models. These models are needed to guide clinical care, appropriately treating those with a chance for favorable outcomes and avoiding unnecessary or painful procedures with little chance for a prolonged quality of life. ML and neural network models–through capturing complex, nonlinear relationships and interaction effects in large datasets–can improve predictions compared to traditional statistical techniques in oncology patients. For example, in brain metastasis patients treated with stereotactic radiosurgery, Oermann et al. demonstrated neural network models with common clinical data such as gender, tumor type, and performance status significantly outperformed regression approaches by nearly ten points in the area under the receiver operating curve [18]. Other models, such as the one developed by Muhlestein et al. to identify high risk patients or those likely to have nonhome discharge, may assist with preoperative counseling and medical optimization [19]. In one of the largest efforts to date, ML models outperformed clinical oncologists predicting 3-month mortality in a multi-institutional cohort of thousands of patients with metastatic cancer [20]. Here, Zachariah et al. used traditional clinical variables easily obtainable in the EHR to build this model with a tree boosting approach [20].

Unfortunately, manual EHR analysis can be tedious. Thus, large language models—using similar techniques to ChatGPT—offer the promise to harness the complex data stored in free text clinical notes. Jiang et al. demonstrated that NLP algorithms could successfully predict a variety of relevant hospital tasks including disposition, comorbidities, and increased length of stay in an “all purpose prediction engine” [21]. Within oncology, Muhlestein et al. and Nunez et al. used this approach to successfully improve nonhome discharge and survival prediction from patient notes [4, 22]. One benefit of these NLP approaches is the relatively simple aspect of data extraction from the EHR [4, 22]. Future approaches will utilize multiple data sources to help guide the patient course from diagnosis to prognosis. For example, Pease and Gersey et al. developed a ‘report card’ that packaged likely diagnoses, genetic alterations, and survival curves in a clinically applicable tool [11]. Applications such as this can provide more information to providers, so that they are best prepared to guide potential conversations with patients and their families, should they seek more information on their clinical course [11]. As data availability increases, these pre-operative tools will lead to objective prognostication and optimal treatment plans that can improve patient care.

### Surgical planning

Advances in CV were amongst the first AI methods to be utilized in the clinical space, and these have shown great promise for planning of intervention and following treatment response. This has been especially apparent in stereotactic radiosurgery (SRS) thanks to sophisticated segmentation algorithms such as the U-net [23]. Recently, Lu et al. used computer vision to automatically identify tumor borders in several different pathologies [24]. This improved contouring accuracy—especially for inexperienced clinicians—and improved efficiency by roughly 30%. In a cohort of patients with only brain metastases, Bousabarah et al. had similar segmentation accuracy but model performance was limited somewhat by lesion size [25]. Performing similar to expert clinicians, Hsu et al’s automated ML pipeline was able to track treatment response [26]. Additionally, similar results were obtained from Peng et al. in the pediatric brain tumor population [27]. Using MRIs from pediatric patients with medulloblastoma and high-grade gliomas, the authors were able to achieve excellent segmentation of tumors both pre- and post-operatively. This allowed for less variability in assessing treatment response in this challenging group of patients. For a more thorough discussion of the use of AI in SRS, see the recent review by Lin et al. [28].

The aforementioned progress in tumor segmentation has also greatly aided planning of neurosurgical tumor resections. For example, Musigmann at al manually extracted 107 features from T1 post contrast MRIs and trained a ML model on just these features to predict the likelihood of gross total resection, which can influence the use of surgical adjuncts like 5-aminolevulinic acid or other intra-operative imaging tools [14]. Importantly, the models used had a level of interpretability that allowed the researchers to determine what features were most predictive of gross total resection, which included expected variables such as tumor location and shape [14]. While such studies show the potential for AI to help guide surgical planning, even more exciting work is being done on automating surgery itself. Currently in development by Tucker et al. is one such tool that uses a robotic laser system to resect brain tumor tissue [29]. It makes use of fluorescence to construct an AI model to help guide the laser to areas of tumor tissue while also incorporating computer vision to help the operator optimize areas for resection [29]. Although the development of these systems is still in its infancy, AI methods are set to potentially revolutionize surgical interventions for brain tumors.

### Intra-operative pathologic classification

While pre-operative tumor genetic predictions aid surgical planning, these predictions are commonly confirmed intra-operatively. A definitive genetic identification is typically made in collaboration with a neuropathologist, which can be laborious, time-intensive, and subject to variability; however, this information can have a rapid impact on surgical outcomes. For example, optimal tumor resection goals can vary between different spinal cord lesions, specific glioma subtypes, and teratoid rhabdoid tumors, which benefit from definitive tumor identification during surgery [30]. Even since the 1990s, neurosurgical teams have harnessed artificial neural networks to enhance identification speed and objectivity [31]. Two recent applications of neural networks provide substantial improvements to histological and molecular tumor identification.

Leveraging advanced microscopy techniques, Orringer et al. introduced a rapid method for tumor imaging, coined stimulated Raman histology (SRH). This technique, which mimics traditional Hematoxylin and Eosin (H&E) histology without requiring staining, allows for tissue structure visualization at a microscopic level [32]. Hollon et al. furthered this approach to build a novel clinical tool by training a convolutional neural network on SRH tumor data [33]. In less than three minutes, this comprehensive clinical pipeline can provide expert-level tumor analysis of pathologic diagnosis and grade during surgery [32, 33].

Compared to histological analyses, molecular-level tumor classification historically required days to weeks for complete analysis, which can provide critical tumor subtype information, like MGMT methylation or IDH mutations. Nanopore DNA sequencing is a rapid technique that has accelerated DNA methylation profile determination, but unfortunately large nanopore-based datasets are nonexistent [30]. To overcome this limitation, Vermeulen et al. developed stimulated nanopore data to train their neural network classifier, Sturgeon [30]. This approach allows Sturgeon to perform a methylation analysis intra-operatively and classify tumor subtypes in less than 90 min [30].

Analysis pipelines harnessing ML have made histological and molecular-based tumor identification readily available within the typical surgical window. However, neural network-based classification is limited by tissue quality [30, 32, 33]. While these tools are readily compatible in clinical settings, the quantity and purity of tumor in resected tissue samples directly influences performance [30, 32, 33]. With reasonable hardware requirements for clinical implementation, this approach can provide a critical supplement when neuropathologists are not readily available [30, 32, 33]. Ultimately, ML based tumor classification produces an accurate, rapid tumor diagnosis, which can positively direct surgical decision-making and improve patient outcomes.

### Surgical skills training and evaluation

Surgical skills are constantly developed and refined through a surgeon’s career. Traditionally, graduated constructive feedback has been the predominant form of learning via a model that is driven by mentored, direct verbal feedback [34–36]. Substantial variability in the effectiveness of this approach is a major limitation to efficient training. The evaluation of surgical skills and improvement of training regimens may be augmented through novel applications of AI, including ML and AR/VR systems. Modern algorithms have emerged as powerful tools that may one day be suited to power personalized training and performance evaluation in surgical practices [37–40]. These technologies can analyze vast datasets of surgical procedures, identify patterns, and optimize training regimens to suit individual learning curves [37–40]. AI-driven simulations provide realistic scenarios, enabling surgeons to hone their skills in a risk-free environment [38]. Additionally, Kiyasseh et al. have used ML algorithms to assess a surgeon’s performance, offering constructive feedback and facilitating continuous improvement [40].

AR/VR systems are only starting to become more available with advanced interfaces that are integrated into surgical fields. By providing a low-risk interface for both the creation and simulation of surgical tasks, VR environments are an increasingly important medium for surgical training. In their systematic review, Chan et al. identified 33 virtual reality systems that simulated multiple surgical procedures while also providing objective feedback, such as force, kinematics, distance to target, and other biologic outcomes (surgical time, blood loss, volume resected) [38]. This automated objective feedback allows for the methodical assessment of skills and evaluation of the skill level between users (i.e. resident versus attending) [38].

Integrating these technologies within surgical training enhances technical skills with the future promising more sophisticated simulations and personalized training modules. The collaboration between medical professionals and technology experts is poised to reshape the paradigm of surgical education, ensuring that practitioners are equipped with the most advanced skills to deliver optimal patient outcomes.

### Pitfalls

Despite the promise of AI tools to improve neuro-oncological care and training, they are limited to the effectiveness of their training. A model or platform reflects their underlying data, and unfortunately, many novel AI tools exist with underlying bias [41]. While this bias is commonly related to racial, ethnic, or socioeconomic factors, there is also risk of small or skewed datasets leading to sampling bias [41–43]. Since it is difficult to understand how many machine learning algorithms reach a decision (i.e. they may be considered ‘black boxes’), it can be challenging to ensure algorithms properly reflect the role that social health determinants play in care and outcomes [41, 42]. Though larger, more robust datasets are slowly emerging in neuro-oncology, most models are still reliant on smaller, potentially unbalanced datasets [14–17, 30, 44]. Therefore, creating robust tools can be more challenging, as algorithms can be susceptible to anomalies, which may worsen their generalizability. Data quality, similarly, varies greatly worldwide and is often worse in under-resourced countries, reducing the accuracy of AI tools trained exclusively on higher quality inputs [43]. Continued efforts to debias AI–with bias mitigation preprocessing, with careful external validation, and with larger, more representative datasets–will be crucial for widespread clinical efficacy [41, 42].

Advances in AI are not healthcare exclusive, and innovations have also increased the possibility of exploitation. For example, working backwards from cranial MRIs, Schwarz et al. reconstructed facial images for patient recognition [45, 46]. While ‘de-facing’ methods exist for altering MRIs, they do not completely protect patients and introduce potential artifacts that influence standard MRI analysis [45]. Therefore, it is important for increased discussion on effective patient consent before including their imaging in larger databases. Along with the continued development of more effective de-identification protocols for data, increased data privacy standards are necessary to facilitate patient protection with growing accessibility to patient data.

### Future

Data consortiums are a growing avenue for centralized creation and dissemination of large, tumor specific datasets. Multi-institutional consortiums, like ReSPOND (Radiomics Signatures for PrecisiON Diagnostics) for glioblastomas and GLASS (Glioma Longitudinal Analysis) for gliomas, have helped create standardized, comprehensive molecular and imaging datasets [47, 48]. Likewise, the Brain Tumor Image Segmentation (BraTS) Challenge is a global competition that makes large MRI datasets widely available and encourages continuous model improvement and evaluation [44]. However, top performing algorithms tended to be isolated to a particular brain region, tumor size, and/or image quality, indicating there is still substantial room for widely generalizable tumor segmentation models [43, 44]. Indeed, many contextual differences exist across health systems, conditions, and patient populations, which increases the complexity of broadly implemented AI tools. Federated learning–where models are shared and updated at different institutions without centralized sharing of data–has emerged as a paradigm to safely enhance training without increased data risk [49]. This approach helps expose models more broadly to diverse data, and along with expanded centralized datasets, has led to vastly improved generalizability of AI tools [49].

Robust AI systems promise to revolutionize neuro-oncological treatment through accurate patient prognostication, rapid tumor segmentation and histopathologic identification, and real-time objective feedback. With growing datasets and increased collaboration for federated learning, these tools will become more effective and clinically integrated. Even now, re-emergence of glioblastomas following initial treatment is predicted with MRI analysis [50–52]. As these models improve, the capability to predict future tumor locations and emergence will drive improved preemptive care. Ultimately, these toolkits along with other AI based tools will lead to holistic, optimized outcomes and enhance the ability of neurosurgeons to treat patients with these challenging diseases.

## Data Availability

No datasets were generated or analysed during the current study.
